# Common Ultrasound Applications for Pediatric Musculoskeletal Conditions

**DOI:** 10.1007/s12178-022-09788-x

**Published:** 2022-08-06

**Authors:** Celina de Borja, Rhonda Watkins, Tiana Woolridge

**Affiliations:** 1 Division of Pediatric Orthopaedics, Department of Orthopaedic Surgery, University of California, San Francisco, 1825 4th Street – 5th Floor, San Francisco, CA 94158 USA; 2 Department of Pediatrics, University of California, San Francisco, San Francisco, USA

**Keywords:** Musculoskeletal ultrasound, Pediatric orthopaedics, Diagnostic imaging

## Abstract

**Purpose of Review:**

To discuss the use of ultrasound for diagnosis and management of common pediatric musculoskeletal conditions through a case-based approach.

**Recent Findings:**

Ultrasound is an essential diagnostic modality in the early detection of developmental dysplasia of the hips and can be used as early as 6 weeks of age when the ossific nucleus has not developed yet. Ultrasound is helpful in diagnosing traumatic injuries such as fractures and intramuscular hematomas, can visualize fracture healing at early stages, and can also be used to guide aspiration of hematomas that can help with decreasing pain and faster recovery. Ultrasound is superior to radiographs in evaluating joint effusions and soft tissue infections or masses and is better tolerated by children compared to other imaging modalities such as magnetic resonance imaging (MRI).

**Summary:**

Ultrasound is an easily accessible, affordable, non-invasive, and radiation-free imaging modality that is well tolerated by children and their families. It can aid in the diagnosis and management of a wide variety of musculoskeletal conditions including developmental, traumatic, and infectious etiologies, as well as in the evaluation of superficial soft tissue masses.

## Introduction

Ultrasound is an imaging modality that is widely used in diagnosing and managing pediatric patients with musculoskeletal conditions. It does not use radiation, it is non-invasive, and it can be performed while the patient is held by the parent for comfort, which makes it well tolerated by children. It is readily accessible in various clinical settings and can be performed at bedside without necessitating sedation, thus, avoiding delays or multiple visits. The clinician performing the procedure can interact with the patient and parent, which allows opportunity for focused history taking. The procedure complements the physical exam, which can help narrow down the differential diagnosis. The clinician can image multiple locations, including the contralateral side, for purposes of comparison, and can perform dynamic assessments [1–5]. Ultrasound can also be used to guide interventional procedures such as injections, aspiration, drainage, or biopsy of musculocutaneous conditions [4]. However, because the quality of ultrasound images is operator dependent and can be limited by patient cooperation, insufficient training of clinicians often challenges optimal utilization of ultrasound in clinical care [2, 3].

Common pediatric orthopaedic conditions, where ultrasound may be valuable in diagnosis and management, include developmental dysplasia of the hip, fracture management, joint effusions, and other superficial musculoskeletal conditions [2]. A high-frequency (10–15 Hz) linear transducer would be appropriate for visualizing small and superficial structures in children. It can describe a lesion’s size, shape, and composition (cystic vs. solid). In addition, applying the Doppler function can be used to evaluate vascularity [1, 2]. This paper will summarize the use of ultrasound for diagnosis and management of some common pediatric musculoskeletal conditions via a case-based approach.

## Developmental

### Case

A healthy full-term female neonate was born via cesarean section due to breech presentation. She presents with a palpable clunk that was noted on Ortolani and Barlow maneuvers. Ultrasound of the hips reveals developmental dysplasia of the hips.

### Developmental Dysplasia of the Hip (DDH)

DDH is a condition that includes a broad spectrum of congenital developmental anomalies of the hip that ranges from acetabular dysplasia, instability, to hip dislocation. It affects 1–2% of infants worldwide, can be associated with certain medical conditions, but can also affect healthy infants due to genetic or intrauterine causes. It commonly presents with a positive Ortolani, Barlow, or Galeazzi on physical exam, which suggest instability; however, stable hips may be silent or asymptomatic during childhood, and are often undiagnosed [2, 6–8]. Late diagnosis increases risk for corrective surgery. DDH is also a known risk factor for early osteoarthritis and hip replacement, with approximately 1/3 of hip replacements performed in patients less than 60 years old resulting from untreated DDH [2, 6].

**Fig. 1:**
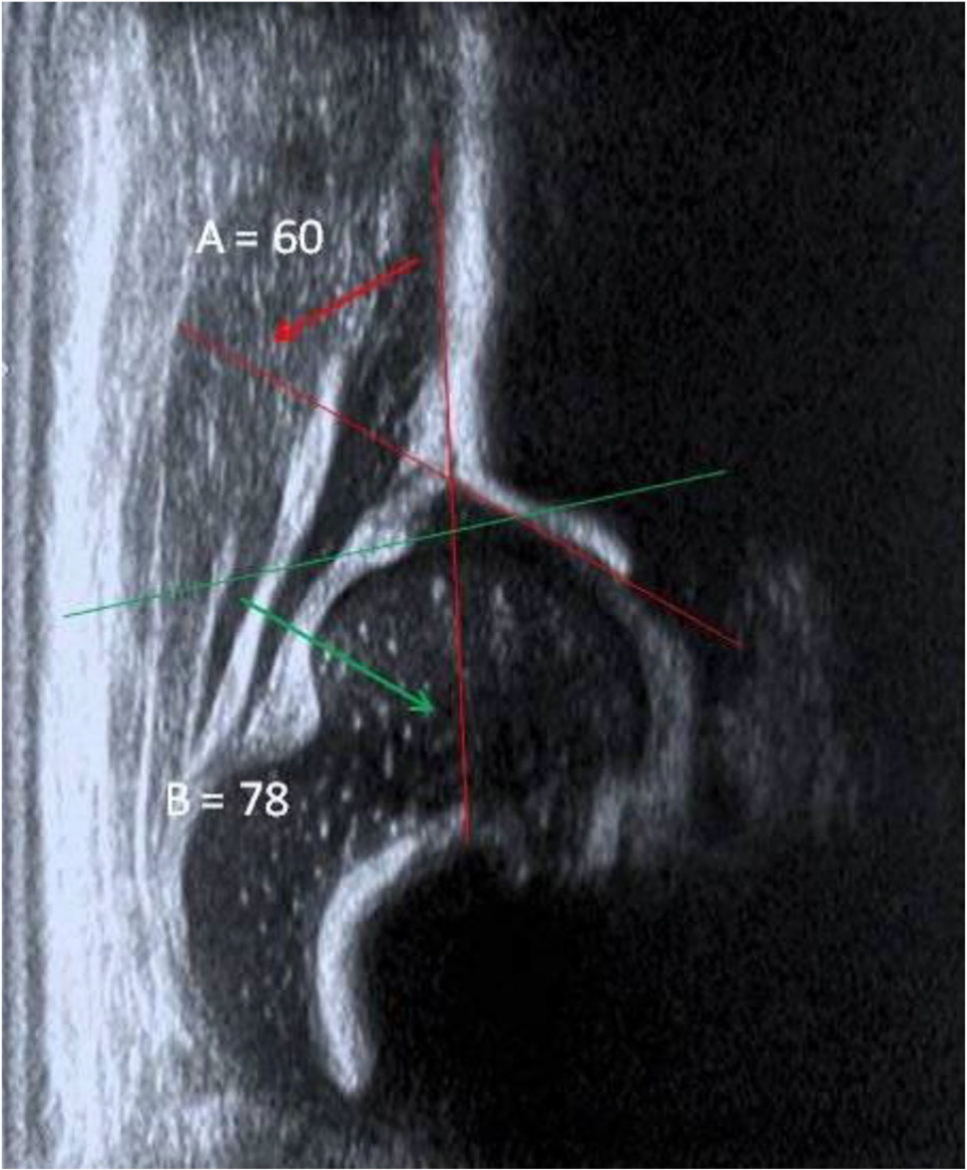
Graf’s method for ultrasound evaluation of developmental dysplasia of the hip shows a normal hip. Case courtesy of Mr Sandeep Hemmadi, Radiopaedia.org, rID: 45301

The sensitivity of the physical exam in detecting hip dislocation is only 37%, which is even lower in hip instability. This can be augmented to 89% by utilizing ultrasound, which allows for capture of dynamic images [2]. Ultrasound has been used in DDH since the 1980s. It is mainly used before 6 months of age when the femoral head ossification has not yet appeared [7, 8]. There are variations in screening practices globally, with some European countries performing universal ultrasound screening for DDH [2, 8, 9]. In the USA, screening is only recommended for those with a positive physical exam or are considered high risk (e.g., breech presentation or pertinent family history), as studies suggest that universal screening only results to increased treatment, which may result in increased iatrogenic complications, and does not necessarily decrease rates of late diagnosis or corrective surgery [1, 2, 6, 8]. Ultrasound evaluation should be performed at 6 weeks of life as evaluations performed within the first 4 weeks may yield false positive results due to physiologic laxity [1, 10]. Graf’s method is most commonly performed due to its high sensitivity, specificity, and reproducibility. This method distinguishes between pathologic hip instability and normal hip laxity based on the static morphology on the coronal plane. It also measures the alpha angle which corresponds to the acetabular bony roof (considered normal if > 60 degrees), and the beta angle that corresponds to the acetabular cartilaginous roof [2, 8, 11]. (Fig. 1). Graf’s method may be limited due to emphasis on procedural technique and high intraobserver and interobserver variability in interpretation, and since ultrasound evaluation is operator dependent, insufficient training amongst clinicians may lead to decreased utilization in the clinical setting. A transcontinental survey of orthopaedic surgeons revealed that 57% of Pediatric Orthopaedic Society of North America (POSNA) members relied on radiology technicians for DDH ultrasound evaluations compared to only 7% of European Orthopaedic Society (EPOS) members [9]. Other less technical methods have also been described. An example is Harcke’s method of dynamic ultrasound that provides objective information and reproducible evaluations that allows for some variation in operator technique. It allows real-time assessment with or without stress maneuvers [8]. These less technical methods might be considered subjective due to operator dependence, but may increase clinicians’ confidence in performing such procedures. A prospective study on the interobserver reliability of an ultrasound-enhanced physical examination revealed very high agreement between learners (pediatric orthopaedic surgeon, orthopaedic resident, and a pediatrician) and an experienced pediatric orthopaedic surgeon in diagnosing abnormal hips (including dysplasia, instability, or dislocation) after completing a 2-hour course, suggesting the feasibility in implementing this skill in orthopaedic and pediatric residency training [7].

Despite extensive literature on the topic, including practice recommendations from the American Academy of Orthopaedic Surgeons (AAOS) and American Academy of Pediatrics (AAP), there is no consensus on best detecting and treating hip dysplasia [6, 8]. A survey of (PONSA) members revealed that although 94% of surgeons agreed that universal ultrasound screening was unnecessary, 68% of surgeons work at an institution that does not endorse a standard care pathway for DDH, and those that did, cited the AAOS guidelines. Additionally, while most surgeons agreed on management, discrepancies still exist due to the large body of literature that have conflicting messages [6]. A Pavlik harness is widely accepted in treating DDH in infants less than 6 months old, where the goal is concentric reduction and acetabular coverage of the femoral head. Its efficacy is inversely proportional to the patient’s age highlighting the importance of early diagnosis and identification of unstable or dysplastic hips [2, 7, 11]. In addition to DDH screening or diagnosis, ultrasound can also be helpful with follow-up. It can be used to monitor development of the acetabulum and ossific nuclei of the femoral head, which can help determine resolution or persistence of hip abnormalities after Pavlik harness. In cases that are diagnosed late or have failed Pavlik harness, ultrasound can also be used to confirm successful closed reduction after spica casting, especially in institutions that do not have access to a CT scan or MRI intraoperatively [2, 8].

## Traumatic

### Fractures

#### Case

A 7-year-old female presents with right wrist pain and swelling after a fall off the monkey bars. She is focally tender over her distal radius with limited range of motion due to pain. Radiographs showed a buckle fracture of the distal radius. Could ultrasound be used for this diagnosis?

Ultrasound has been documented as a diagnostic tool for pediatric fractures as early as 1988, when a case series of 41 newborn infants with a suspected clavicle fracture following traumatic delivery were accurately diagnosed in all cases [12]. It has been reported for use in fractures of the skull, nasal bones, facial bones, clavicle, elbow, forearm, metacarpals, hip, foot, and ankle [13]. Ultrasound is well tolerated by children, easily accessible, more affordable than x-rays, and decreases the number of serial radiographs associated with fracture management. Furthermore, an estimated 82.8% of radiographs performed in injured children show no fracture [14]. Despite this, ultrasound is not yet routinely used in practice for fracture care.

**Fig. 2:**
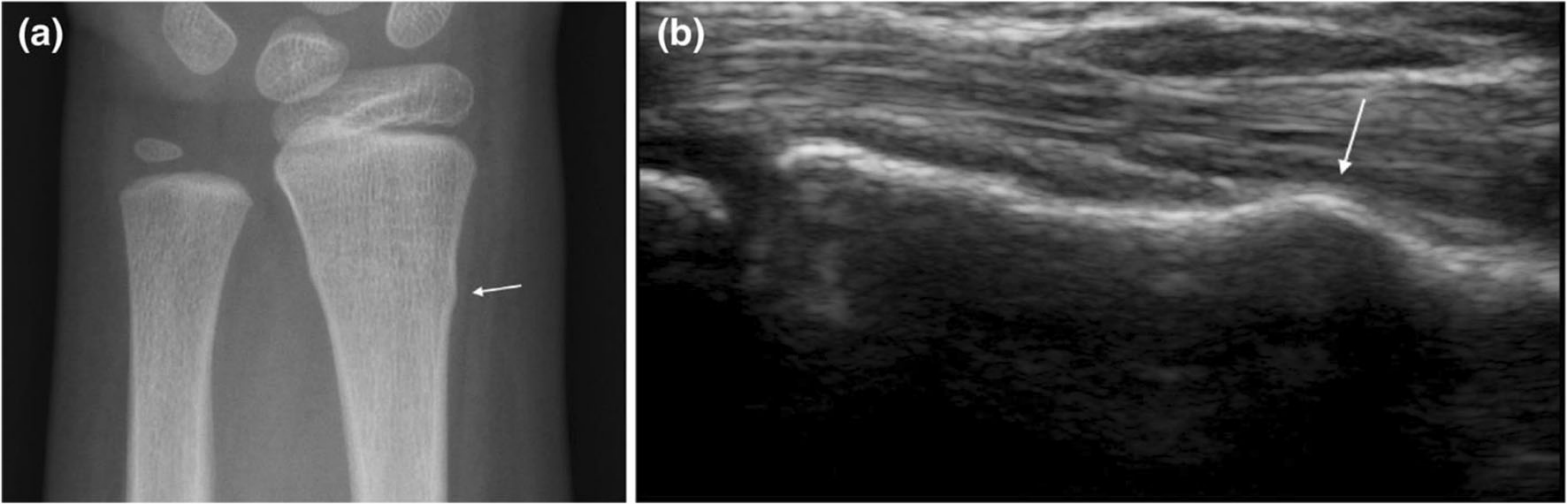
**a** Radiograph of a distal radius type A buckle fracture (arrow). **b** Ultrasound image of a distal radius buckle fracture (arrow) on the same patient (Snelling, 2018). Reprinted with permission from Wiley Online Library [19]

Under ultrasound, fractures can be seen as echogenic disruptions of the cortical line, usually with anechoic or hypoechoic fluid collections that represent hematoma formation near the fracture site [15] (Fig. 2a, b). Several studies have shown that ultrasound could be considered as a proper and accurate screening method for diagnosing extremity fractures in pediatric trauma patients, but sensitivity and specificity reports vary. A recent prospective study of 40 pediatric patients, admitted for limb trauma, and evaluated with both ultrasound and radiographs found that ultrasound was faster and had overall 100% sensitivity, specificity, and accuracy in detecting fractures [16]. A systematic review found that the sensitivity and specificity of ultrasound in diagnosing radiographically occult scaphoid fractures ranged from 77.8 to 100% and from 71.4 to 100%, respectively [17]. In another pediatric study of 48 patients with forearm fractures confirmed by radiographs, ultrasound showed 97% sensitivity and 100% specificity in detecting pediatric forearm fractures, and was able to correctly identify the fracture type and location in 95% of patients [18].

Ultrasound has also been reported in the evaluation of fracture healing. There are three major phases of fracture healing: first, the reactive phase in which there is an influx of inflammatory cells, followed by granulation tissue formation. Second, is the reparative phase, in which calcifications are laid down in the callus, leading to formation of cartilage callous. Lastly, in the remodeling phase, lamellar bone deposition replaces the hard callus. Sonographic signs of healing can be identified on ultrasound in all phases of the healing process and as early as 1–2 weeks post fracture [20, 21]. This allows for detection of healing before radiographs. Ultrasound findings of fracture healing include a mound of bridging callus over the superficial cortex on grayscale sonographic images, hyperemia of the adjacent soft tissues on color Doppler imaging, and hyperechoic tissue filling the fracture gap and obscuring visualization of intramedullary implants [20]. In addition, ultrasound has also been reported useful for fracture reduction. A study of 100 pediatric patients with forearm fractures found that ultrasound-guided fracture reduction was successful in 82 out of 100. They concluded that ultrasound was useful in determining adequate fracture realignment, but not in identifying need for further reduction [22].

Ultrasound use for fracture care is limited in open fractures, patients who are unable to localize pain in cases of polytrauma, or when a skeletal survey is needed [13]. It cannot be used in the evaluation of a splinted or casted fracture site and may also be less successful in the diagnosis of non-displaced fractures. In a study of 100 pediatric clavicle fractures, ultrasound was unable to identify two fractures in this population that were visible on radiographs despite 95% sensitivity and 96% specificity for diagnosing clavicle fractures [23].

### Intramuscular Hematoma

#### Case

A 17-year-old male baseball athlete presents with right knee pain and swelling after his thigh was hit by another player’s helmet 3 days ago. On exam, he had bruising over the medial thigh with a palpable, tender mass. Bedside ultrasound showed a well-circumscribed hypoechoic mass with no Doppler signal in the vastus medialis, consistent with a hematoma.

**Fig. 3:**
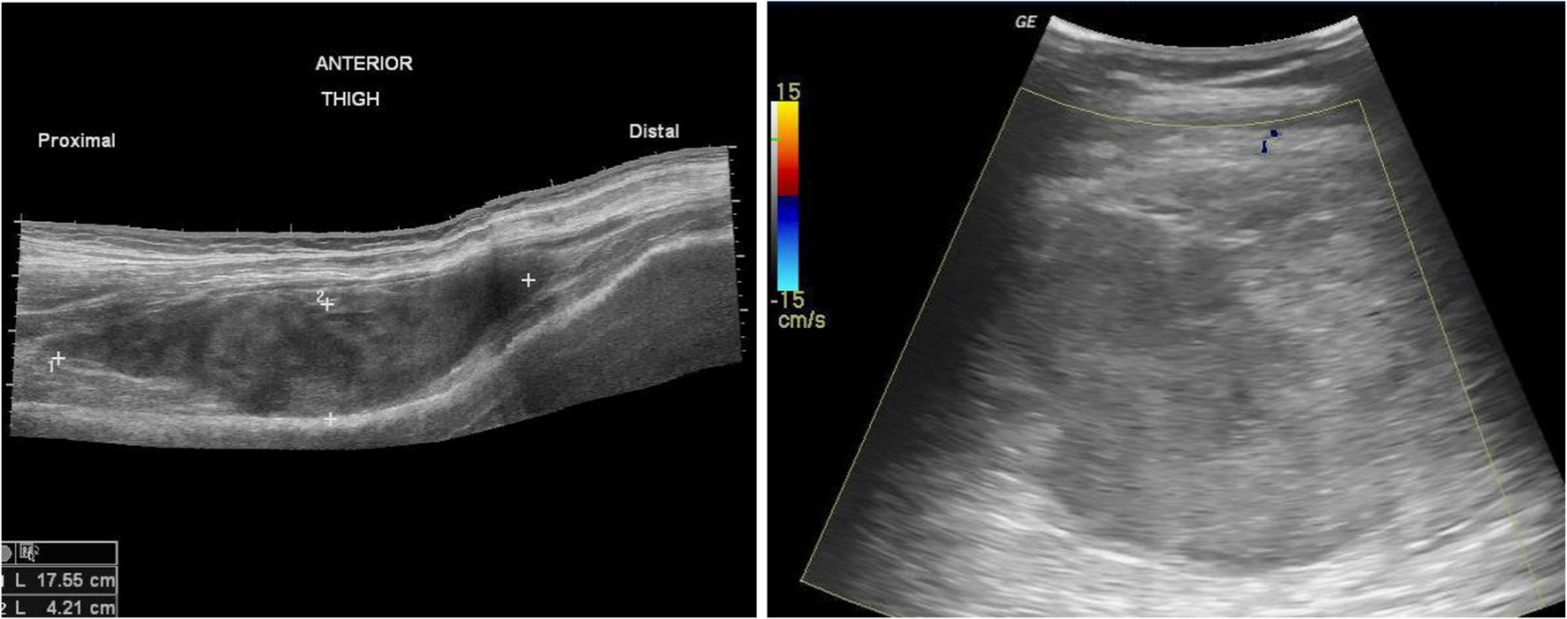
**a** Long axis view of the anterior thigh shows a well-defined area in the vastus intermedius muscle consistent with a hematoma. **b** Doppler short axis view of the lesion shows no vascularity. Case courtesy of Dr Maulik S Patel, Radiopaedia.org, rID: 24042

Soft tissue masses are commonly seen in the pediatric population, with a wide differential including synovial cyst, hematoma, abscess, foreign body, and hemangioma. Ultrasound can be helpful in the characterizing lesions and is even considered the modality of choice for small, superficial lesions, or when the clinical history strongly suggests a specific diagnosis such as an abscess, hematoma, or lymphadenitis. Grayscale images can determine echogenicity compared to surrounding normal tissue and the architecture of the mass, whereas color Doppler can show any vascularity within the mass. Furthermore, ultrasound has the added benefit of assessing compressibility and demonstrating blood flow without contrast administration [2, 24, 25] (Fig. 3a, b).

Intramuscular hematomas may occur from trauma that results in disruption of the muscle fibers with subsequent intramuscular bleeding and hematoma formation. On ultrasound, a soft tissue hematoma may be hyperechoic in the acute phase, but is generally hypoechoic without internal Doppler signal, unless there is active bleeding [25]. Ultrasound can also be used in treatment of hematomas, allowing for image guided aspiration, which can decrease pain and allow for faster return to activity [26].

## Infectious

### Case

A 6-year-old boy presents with an atraumatic limp and inability to weight bear on his right lower extremity for two days. He is febrile, ill-appearing, and has his right leg resting in a position of flexion, abduction, and external rotation. Laboratory studies reveal elevated white blood cells (WBC), erythrocyte sedimentation rate (ESR), and C-reactive protein (CRP), and ultrasound evaluation of his hip confirms presence of an effusion.

### Joint Infections

The differential diagnosis for pediatric hip pain is broad and includes transient synovitis, septic arthritis, osteomyelitis, slipped capital femoral epiphysis (SCFE), juvenile idiopathic arthritis (JIA), Legg-Calve-Perthes’ (LCP) disease, pyomyositis of the muscles surrounding the hip, bony tumors, fracture, hemarthrosis in patients with a clotting disorder, or referred pain from abdominal pathology such as appendicitis with a pelvic abscess [27]. A thorough history, physical exam and basic laboratory studies can help determine the appropriate imaging modality.

**Fig. 4:**
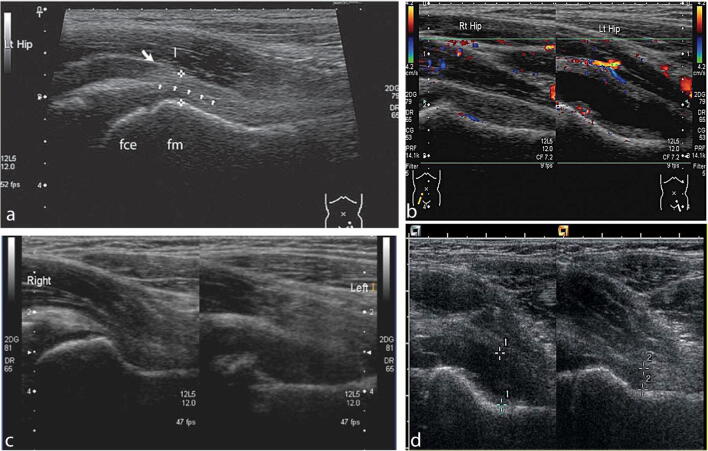
**a** Normal anatomy of the anterior hip joint capsule. fce, femoral capital epiphysis; fm, femoral metaphysis; between cursors, both layers of joint capsule (hyperechoic to muscle); I, iliopsoas muscle; small arrows, echogenic interface between joint capsule layers (Crow, 2015). Reprinted with permission from Wiley Online Library [30]. **b** Normal right hip compared to the left hip with an effusion. Hyperemia of the soft tissues is evident on the left side. The machine settings are kept the same for comparison (Crow, 2015). Reprinted with permission from Wiley Online Library [30]. **c** Lytic lesion in the metaphysis of femoral neck with effusion, consistent with osteomyelitis (Crow, 2015). Reprinted with permission from Wiley Online Library [30]. **d** Septic arthritis of the right hip joint. Septations are seen within the fluid (Crow, 2015). Reprinted with permission from Wiley Online Library [30]

Imaging the hip joint can be performed using ultrasonography, radiography, computed tomography (CT), or magnetic resonance imaging (MRI). Shahid et al. recommend utilizing sonography as a first line investigation in a young limping child with a swollen or painful joint to determine whether an effusion is present [28]. The anterior hip joint capsule is brought into view to determine whether there is a separation between the femoral neck and iliopsoas muscle, which may indicate the presence of an effusion [29] (Fig. 4a, b). Radiographs are insensitive for joint effusion, while MRI often requires anesthesia for younger patients and is susceptible to metal and motion artifact [27]. Ultrasonography has the benefit of being more readily available and more affordable than the other imaging modalities, without the risk of exposure to radiation. In addition to evaluating for an effusion, sonography can also be used to identify bone irregularities in the setting of osteomyelitis or LCP [30] (Fig. 4c).

There is significant overlap between the clinical presentation of transient synovitis and septic arthritis [29]. Early diagnosis and treatment of septic arthritis is important as bacterial endotoxins and the body’s inflammatory response can lead to joint damage [28]. The Kocher criteria (refusal to bear weight, fever > 38.5 °C, WBC > 12,000 cells/mm^3^, and ESR > 40mm/h) can help differentiate between transient synovitis and septic arthritis [31]. Ultrasonography is useful for distinguishing between septic arthritis and transient synovitis in pediatric patients with hip pain: the finding of predominant synovial thickening relative to joint effusion thickness at the anterior femoral recess is a statistically significant predictor of septic arthritis, in addition to body temperature > 38.5 °C and elevated CRP [32]. (Fig. 4d). However, it is important to recognize that effusion may not be appreciated on ultrasound within the first 24 hours of symptom onset of septic arthritis [27].

The therapeutic value of ultrasonography includes joint fluid aspiration to decompress the joint and reduce pain [30]. Testing of aspirated joint fluid from an infected joint is important in choosing appropriate antibiotic coverage.

#### Case

A 9-year-old girl presents with left leg swelling after visiting a camp site where she acquired multiple bug bites. She is afebrile and well appearing. On the lateral aspect of her left leg, she has an indurated, non-fluctuant, 4 × 3 cm area of erythema that is tender to palpation. Point-of-care ultrasound (POCUS) shows a focal fluid collection within the area of erythema.

### Skin and Soft Tissue Infections

It is important to differentiate between non-purulent cellulitis and purulent collections such as abscesses, carbuncles, and furuncles, as cellulitis can be treated with antibiotics, while purulent collections require incision and drainage [33]. Failure to appropriately diagnose and manage purulent collections can prolong symptoms and lead to spread of infection to bone or through the bloodstream [34].

**Fig. 5:**
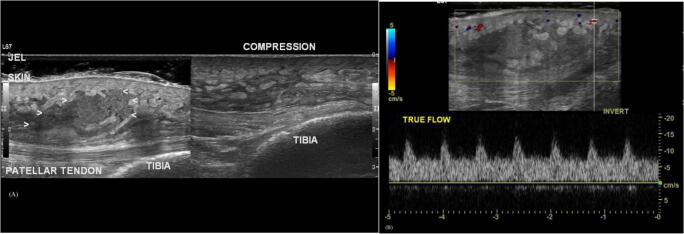
**a** There is a compressible subcutaneous collection in the infrapatellar anterior knee. There are mobile echoes in it. **b** Doppler shows that there is local hypervascularity. Case courtesy of Dr Maulik S Patel, Radiopaedia.org, rID: 50551

The absence of fluctuance on physical examination does not rule out the presence of an abscess. Sivitz et al. demonstrated that only half of patients with an abscess in their study had fluctuance on physical examination [35]. Using POCUS to evaluate a soft tissue infection can improve the sensitivity for detecting the presence of an abscess, particularly in cases where the physical exam is unequivocal [33, 36, 37]. A study by Lam et al. showed that POCUS changed treatment plans in approximately a quarter of cases. Additionally, physicians noted that ultrasound helped guide the incision and drainage (I&D) and can help facilitate parental and patient “buy-in” of an invasive I&D procedure [38] (Fig. 5a, b).

## Superficial Soft Tissue Mass

### Case

A 2-year-old boy presents with a non-painful, round lump on the dorsal aspect of his left wrist that has been noticed over the past 6 months. On physical exam, the lump is firm, movable, compressible, non-erythematous, and nontender to palpation.

Ultrasound can be very useful in evaluating superficial soft tissue masses. It can be used to describe the size, echogenicity that may help differentiate a lesion’s composition (cystic vs. solid), its vascularity, and position in relation to other anatomic structures [2]. According to a 5-year retrospective review by Shah and Callahan, synovial cysts and vascular malformations are the most commonly diagnosed palpable musculocutaneous extremity lesions in children [5].

### Synovial Cyst

**Fig. 6:**
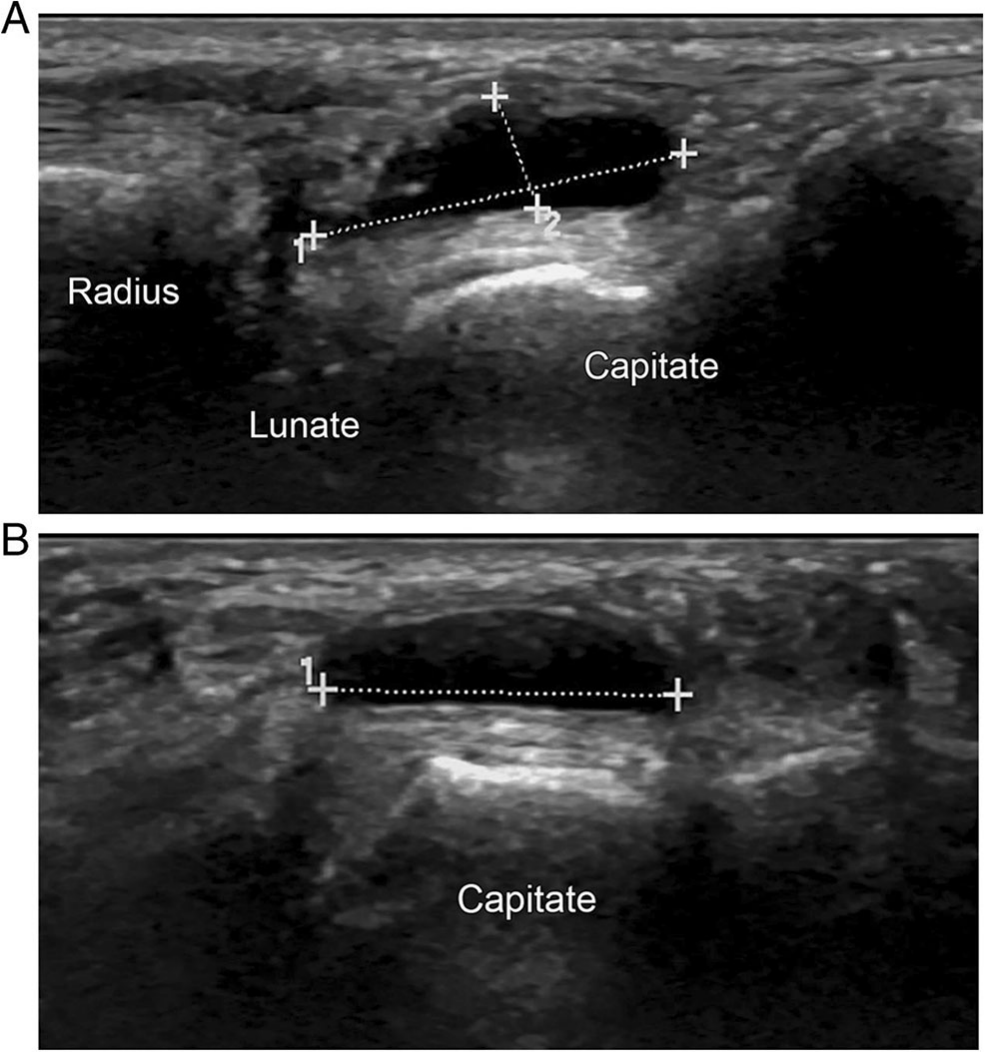
Dorsal ganglion cyst. Ultrasound images of dorsal wrist in (**A**) sagittal and (**B**) transverse planes show anechoic multilobular ganglion cyst (between cursors). Right side of image **A** is distal (Zhang, 2018). Reprinted with permission from Wiley Online Library [41]

Synovial cysts are fluid-filled structures with synovial cell lining, which arise from herniations from synovial membrane through the joint capsule. They usually present as round, firm, compressible and movable superficial lesions that may be painful or pain-free, without skin color changes [39, 40]. They may occur at any site, but are most commonly found on the dorsum of the wrist, and are considered the most common benign soft tissue of the hand or wrist [1, 40]. Amongst the different soft tissue masses, ultrasound has highest accuracy in diagnosing cystic masses [2] (Fig. 6a, b). An ultrasound evaluation may reveal a simple anechoic lesion, or a lesion with septations or loculations, without any solid components or vascularity. Ultrasound may also visualize the synovial cyst’s extension into the tendon sheath or joint [1, 5, 40]. Symptomatic cysts may be aspirated or surgically excised [1, 40]. Recurrence rates in children following surgical excision range from 2.8 to 50%, which are higher than those seen in the adult population [39]. Factors that have been found to predict recurrence include cyst location (with those located on the dorsal wrist having higher recurrence than non-wrist locations), older age, and symptomatic cysts [40]. Ultrasound guidance may be used for cyst aspiration. A retrospective review of percutaneous ultrasound-guided ganglion fenestration in children performed at a tertiary referral academic center revealed that 98% of children (3 to 18 years old) were able to tolerate the procedure without sedation. These procedures were performed through the help of child life specialists and use of a topical anesthetic cream. The procedures included intra-remnant steroid instillation, followed by bracing with activity restrictions for 4 weeks, and resulted in no complications such as infection, skin depigmentation, and dimpling. In addition, none of the participants reported using narcotics for pain relief after the procedure. The overall recurrence was 28.9% at 3weeks to 10 months post procedure. The study concludes that ultrasound-guided ganglion fenestration may be a considered as a safe and minimally invasive alternative to surgical excision in children [39].

### Vascular Malformations

**Fig. 7:**
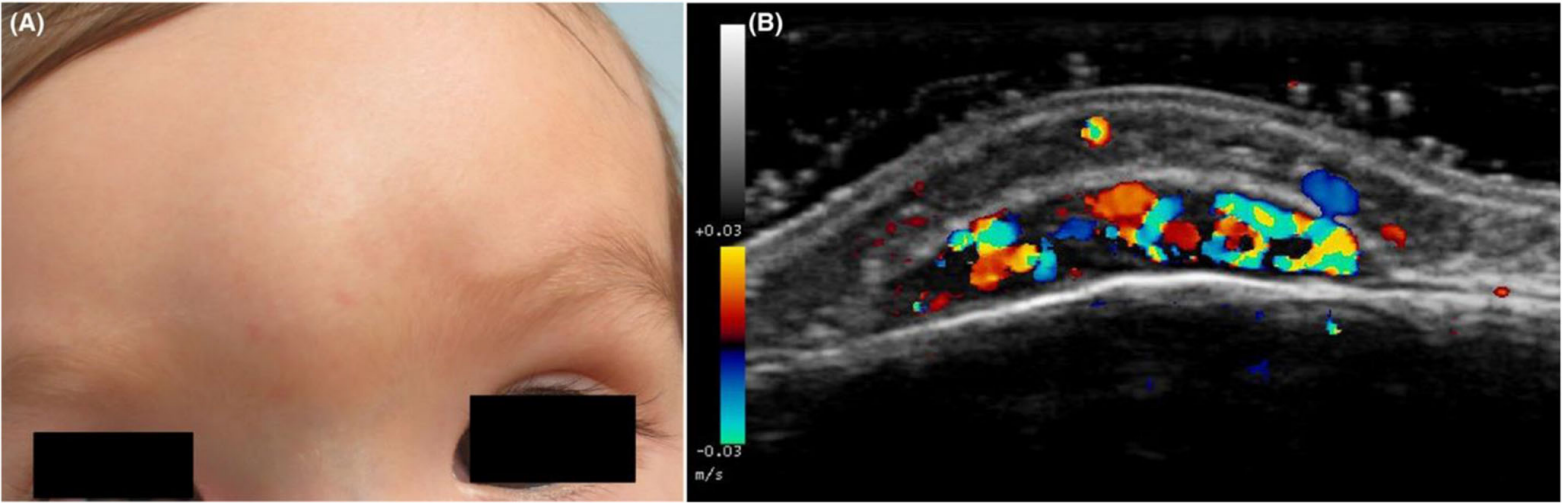
Infantile hemangioma. **a** Clinical picture shows a skin-colored soft mass with some subtle superficial telangiectasias on the glabella of a 7-month-old twin boy. **b** High-frequency ultrasonography (18 MHz) image (Doppler mode) reveals a subfascial hypoechoic well-defined hypervascular lesion with turbulent blood flow. The underlying frontal bone appears unaffected (Bandera, 2019). Reprinted with permission from Wiley Online Library [42]

Vascular malformations are another group of commonly encountered palpable and visible abnormalities in children’s extremities. They usually present as soft, non-painful, superficial lesions, without well-defined borders, that may be associated with skin color changes [5]. They can be categorized as low flow (lymphatic, venous, capillary, or mixed malformations) or high flow (arteriovenous malformation and fistula) lesions [1]. Ultrasound imaging will reveal a group of anechoic channels instead of a solid mass or tumor. High-frequency Doppler ultrasound can help differentiate flow (high vs. low). Low flow lesions such as venous malformations are compressible, have well-defined hypoechoic spongy masses that may show phleboliths, while lymphatic malformations appear as cystic spaces with septations that are fluid-filled, and usually have no blood flow. High flow lesions such as arteriovenous malformations will show arterial and venous blood flow with arteriovenous shunts, without a solid mass that is usually seen in hemangiomas [5, 42] (Fig. 7a, b).

## Conclusions

Ultrasound is an easily accessible, affordable, non-invasive, and radiation-free imaging modality that is well tolerated by children and their families. It can aid in the diagnosis and management of a wide variety of musculoskeletal conditions including developmental, traumatic, and infectious etiologies, as well as in the evaluation of superficial soft tissue masses. Ultrasound utilization may be limited by operator dependence and variations in interpretation. Increasing training amongst clinicians may improve ultrasound utilization in appropriate clinical settings [1–5].
